# Supplementary material for: Gate-controlled reversible rectifying behaviour in tunnel contacted atomically-thin MoS2 transistor
Source: Nat Commun. 2017 Oct 17;8:970. doi: 10.1038/s41467-017-01128-9 (PMC5645421; doi:10.1038/s41467-017-01128-9)
Supplement: Supplementary file 1 — Supplementary Information [file 41467_2017_1128_MOESM1_ESM.pdf]

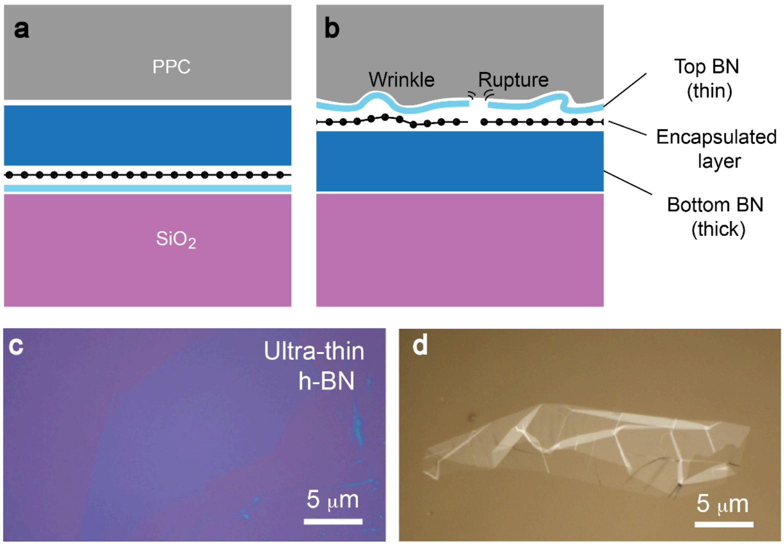

Supplementary Figure 1. **Schematics of conventional vdW stacking process**. Thin layers of h-BN are used as bottom **(a)** and top **(b)** layer, respectively. When the top layer is ultra thin, chances of having wrinkles and ruptures increase significantly **(c)**-**(d)**.

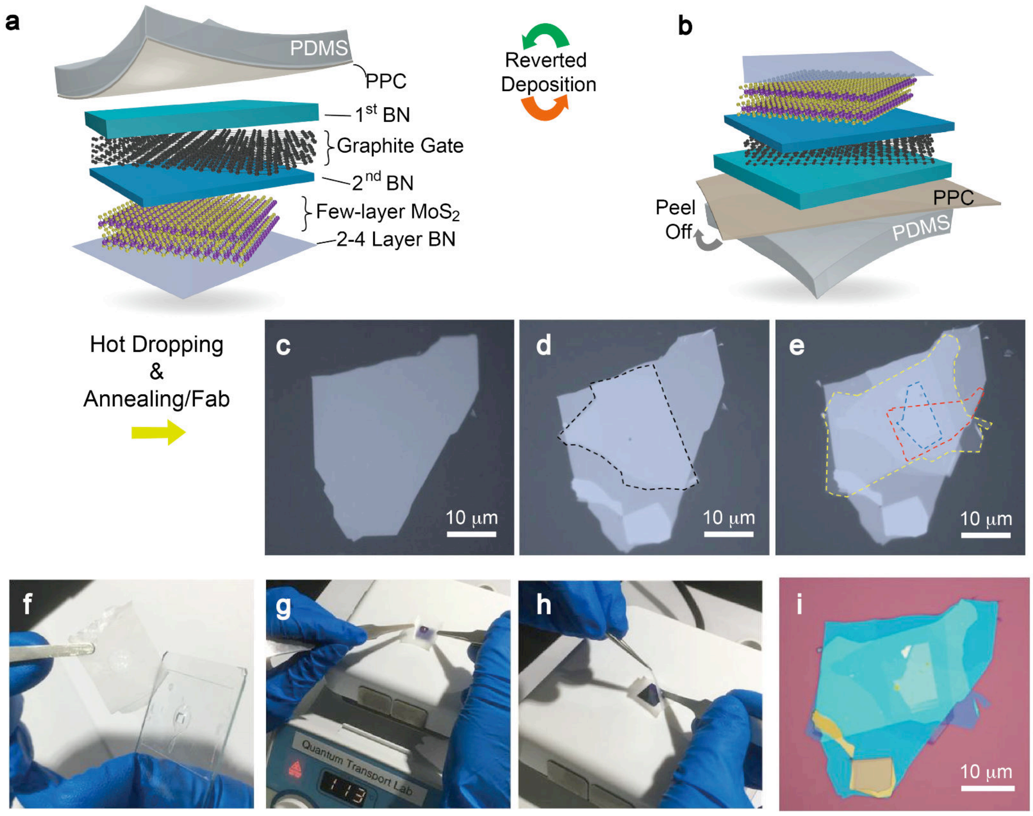

Supplementary Figure 2. Work flow of reverted-vdW stacking process. (a)-(b) Cartoon illustrates in an art view of the workflow of reverted vdW staking method for fabricating vertical hetero-structures with high quality wrinkle-free ultra-thin top layer. **(c)** The top layer of the first thick h-BN picked up by PPC. Subsequent layers will be dry-picked one after another by van der Waals force. **(d)** A graphite gate with thickness of about 4~6 nm was used, to improve the gate efficiency and uniformity. The graphite flake is highlighted with black dashed line. **(e)** A few-layered MoS_2 which is sandwiched by a thick (~10 nm) h-BN and thin (2-4 layer) h-BN, respectively. The three flakes (thick h-BN, MoS_2 and thin h-BN) are highlighted with yellow, blue and red dashed line. **(f)** PPC with the vdW stack is flipped upside down, and peeled off with care from the PDMS substrate. **(g)** Landing the peeled PPC (with vdW stack facing up) on a hot SiO_2/Si substrate, at about 100℃. **(h)** By removing the surrounding Scotch tape, the stack will be left on hot wafer and float on the PPC film. **(i)** The final stack when underneath PPC was completely removed by vacuum annealing at 350℃ for 20 min.

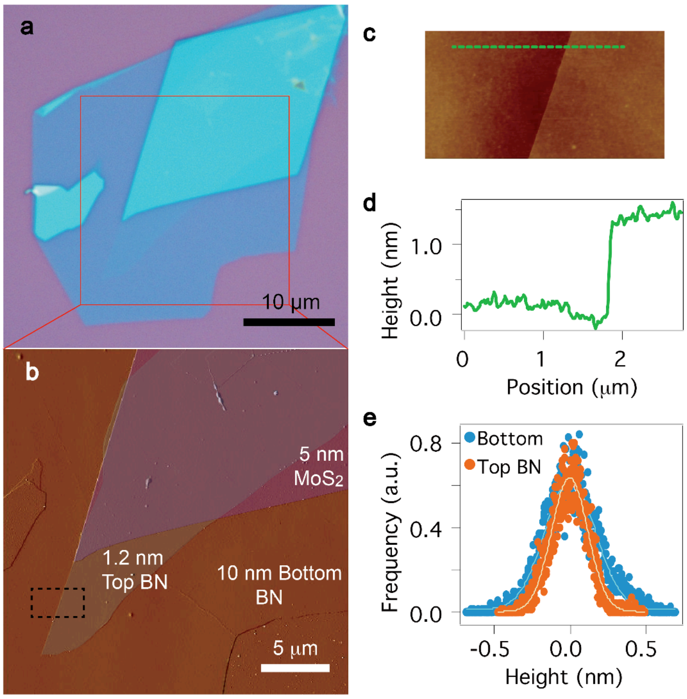

Supplementary Figure 3. **Morphology of revert stacked vdW heterostructure. (a)** Optical micrograph of a typical vertical vdW heterostructure, with ultra-thin h-BN on top. Red-boxed area in **a** is scanned in **(b)** with Atomic Force Micrograph (AFM) amplitude error image. **(c)** Morphology image of black-boxed area in **b**, with a green dashed line height profile plotted in **(d)**. **(e)** A statistics of roughness in top and bottom h-BN in the area shown in **c**, respectively. Roughness of top-BN is smaller than 0.2 nm.

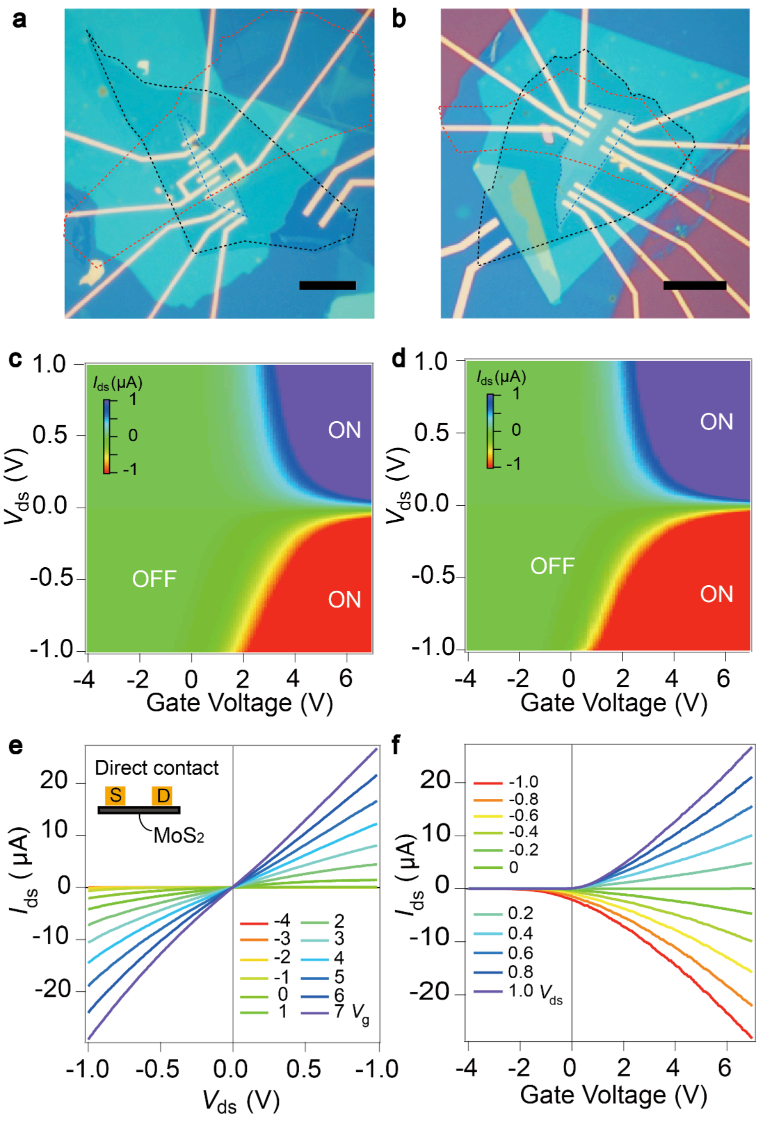

Supplementary Figure 4. **Normal contacted MoS_2 transistors**. **(a)-(b)** Optical micrographs of two measured devices. Graphite, MoS_2 and top BN are highlighted with black, blue, and red dashed line, respectively. Scale bars are 10 μm. **(c)-(d)** Color map of output curves (I_{ds} vs V_{ds}) at different gate voltages for the corresponding direct metal-contacted MoS_2 FETs in **a**-**b**, respectively. **(e)-(f)** Line cuts in **c**-**d**, with output curves along fixed gate voltages, and transfer curves (I_{ds} vs V_g) along fixed bias voltage, respectively.

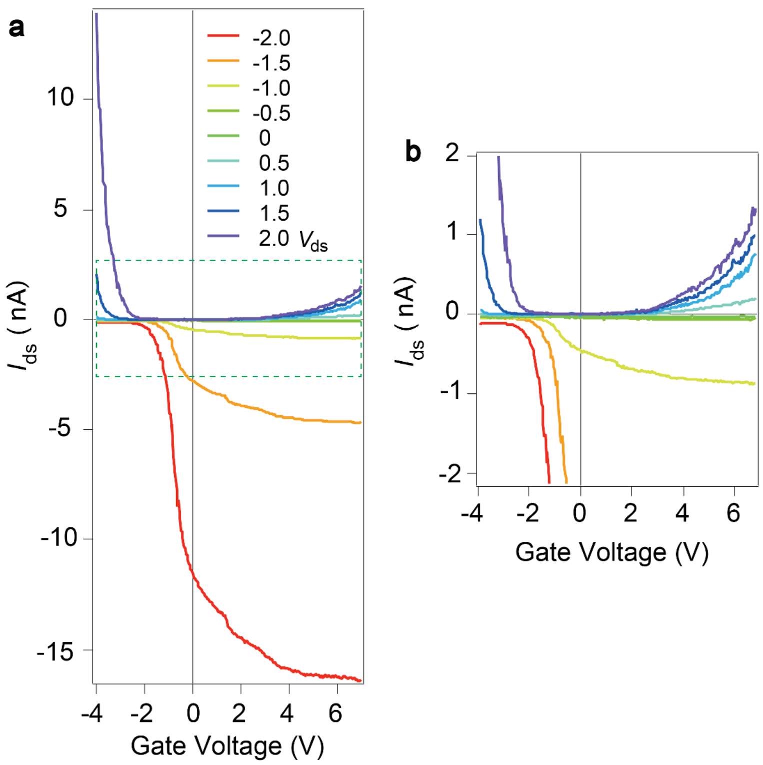

Supplementary Figure 5. Transfer curves of MoS_2 TC-FET. (a) Transfer curves cut from Fig. 2a in the main text along fixed bias voltages V_{ds}. **(b)** is a zoomed-in view in the green dashed box in **a**.

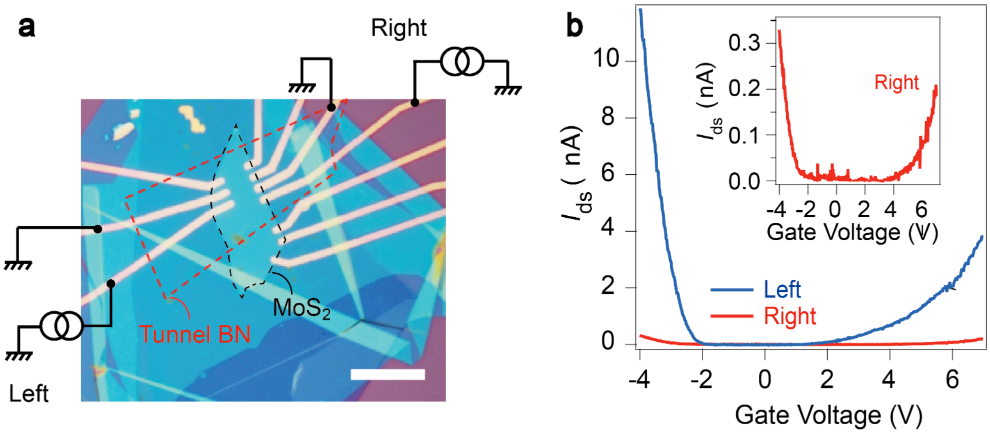

Supplementary Figure 6. **Contact area and dimension of MoS_2 TC-FETs. (a)** Optical micrograph of the device. The top tunnel h-BN is highlighted with red dashed line, and MoS_2 is highlighted with black dashed line. Scale bar is 10 μm. **(b)** The I_{ds}-V_g curves of left tunnel electrodes (blue solid line) and right tunnel electrodes (red solid line) in (a) along +2V bias voltage V_{ds}. The inset of **b** is the zoomed-in image of the red solid line in **b**.

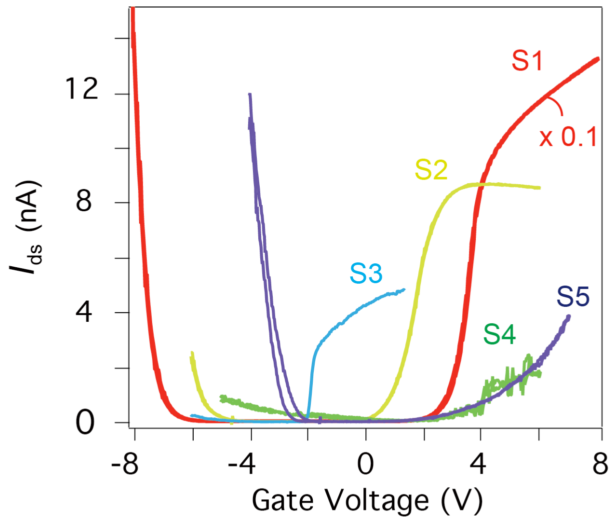

Supplementary Figure 7. **Ambipolar field effect in MoS_2 TC-FETs.** Ambipolar field effect curves observed in different MoS_2 TC-FET samples at V_{ds} = +2 V. S1-S5 denotes the index of each sample.

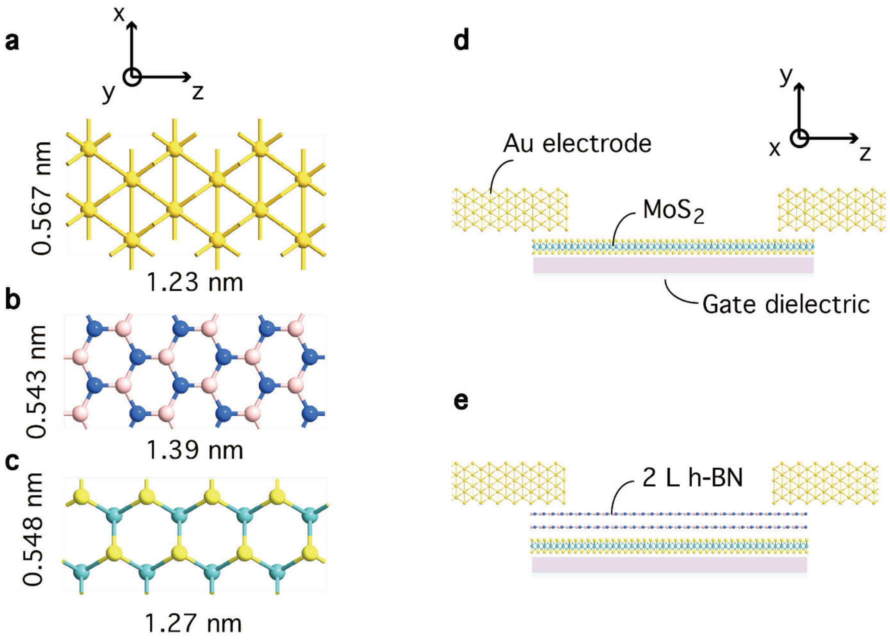

Supplementary Figure 8. **Structural setup of the simulation model. (a)-(c)** Super cell structures of Au/BN/MoS_2 used in the simulations. **(d)-(e)** Theoretical model for Au-contacted MoS_2 FET, and the TC-FET, respectively.

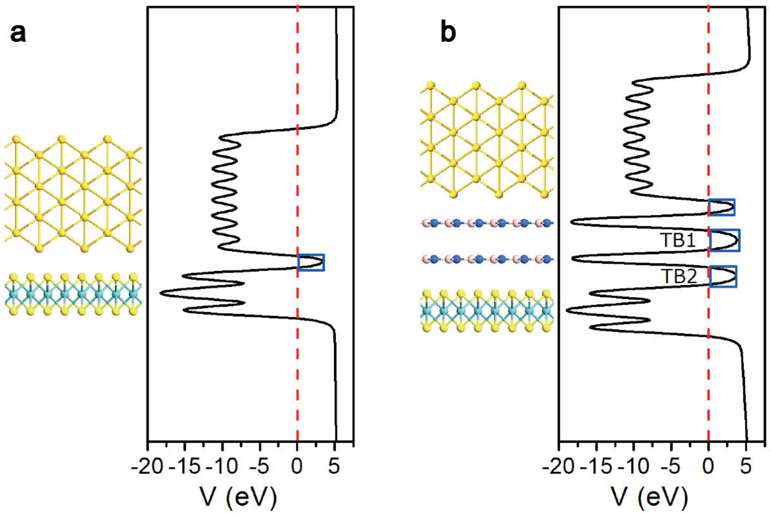

Supplementary Figure 9. Simulated tunnel barrier profiles. Side views of the average electrostatic potentials along *y* direction for each model. **(a)** Normal contact, and **(b)** 2-layered h-BN tunnel contact. The Fermi level is set to zero. The blue rectangular boxes represent the tunnel barriers because of the vacuum gap.

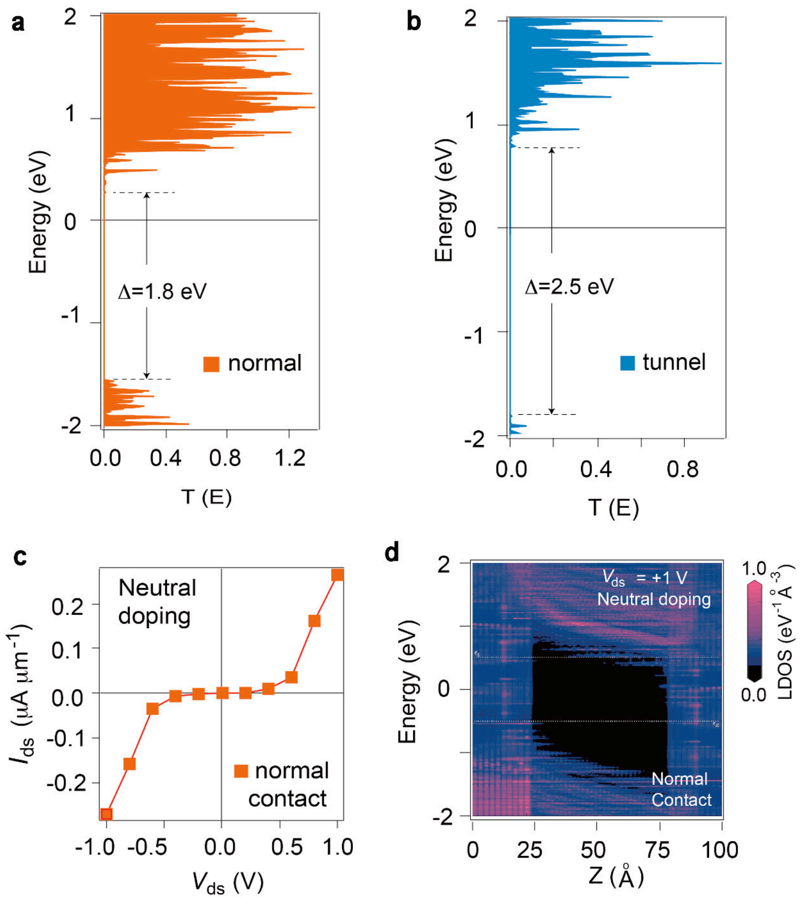

Supplementary Figure 10. **Transmission and device behavior. (a)-(b)** Transmission spectra of normal contacted MoS_2 FET and MoS_2 TC-FET at zero bias, respectively. **(c)** Simulated *IV* curve at V_g=0 V, and **(d)** projected local density states (PLDOS) at V_{ds}=+1 V for normal contacted MoS_2 FET.

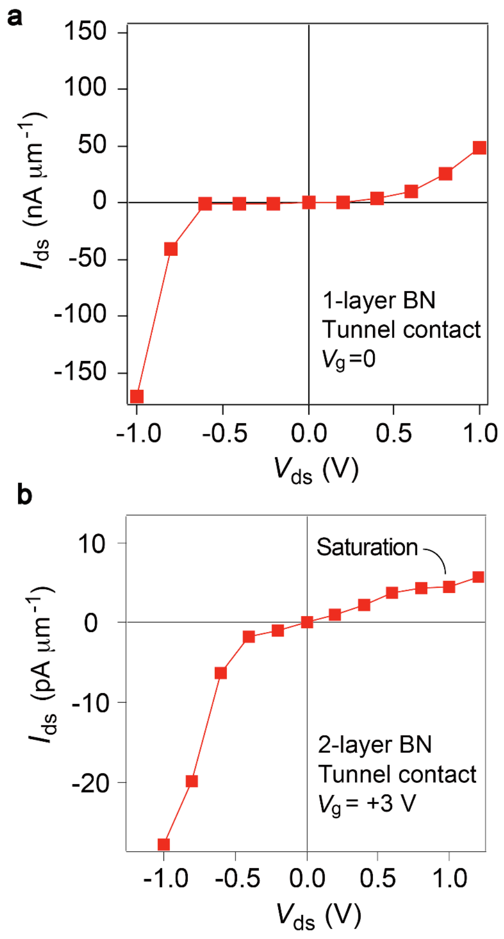

Supplementary Figure 11. Simulated *IV* curves for MoS_2 TC-FETs. *I-V* characteristics predicted by *ab initio* simulations for **(a)** 1-layered h-BN tunnel contacted FET under gate voltage $V_g = 0$ V, and **(b)** 2-layered h-BN tunnel contacted FET under gate voltage $V_g = 3$ V.

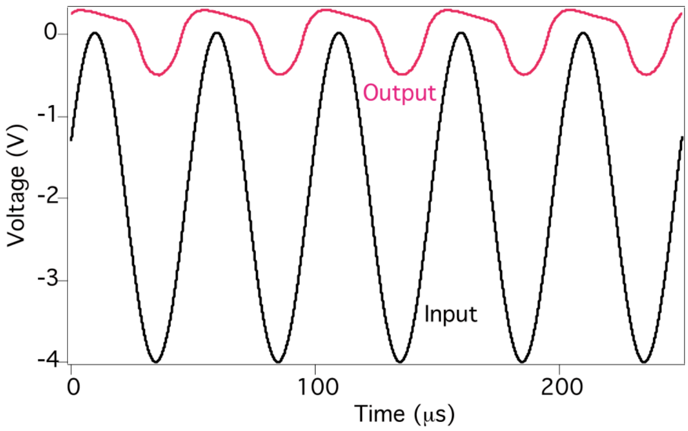

Supplementary Figure 12. **Rectifying behavior of MoS_2 TC-FET at 20 kHz.** Output wave of the MoS_2 TC-FET with an input wave at the frequency of 20 kHz.

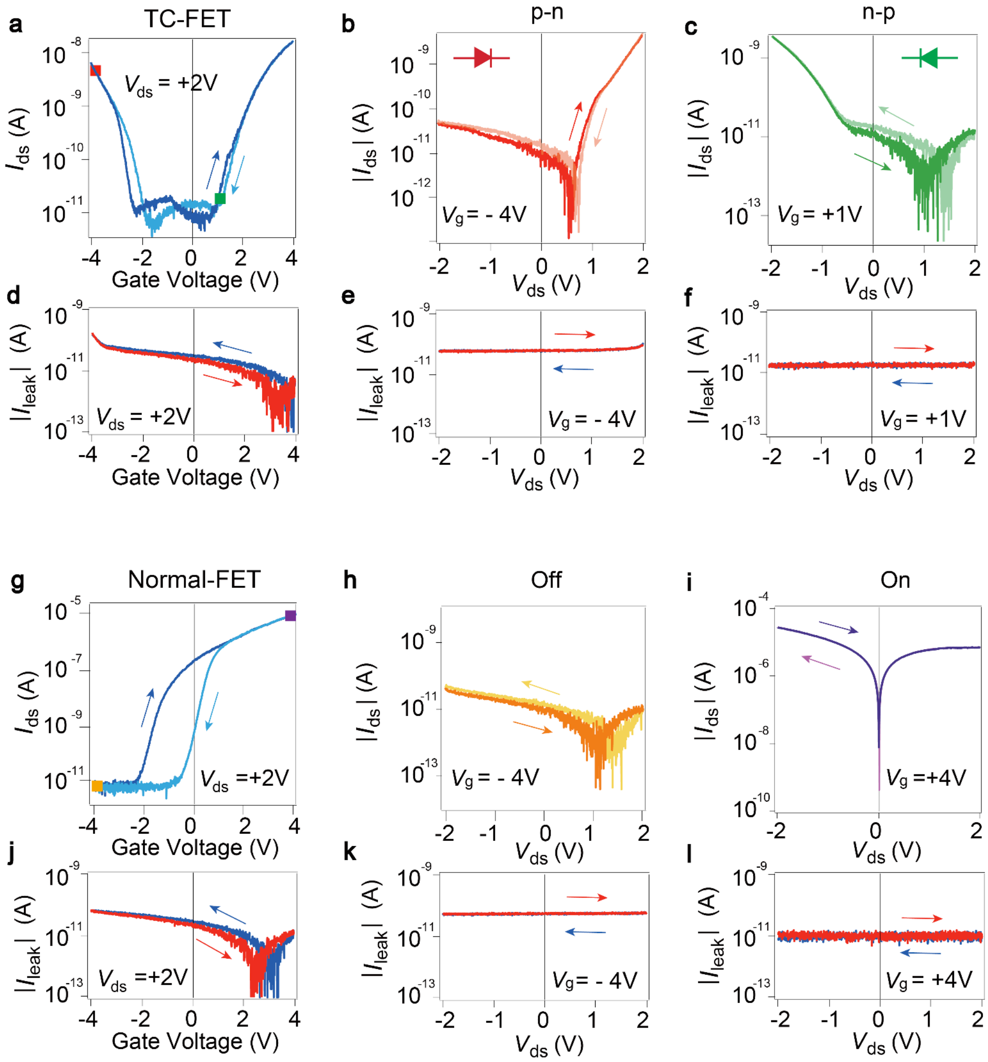

Supplementary Figure 13. **Hysteresis measurements for MoS_2 normal and TC-FETs. (a)-(c)** Log scale plots of hysteresis measurements of field effect and *IV* curves for MoS_2 TC-FET, with **(d)-(f)** the gate leakage current I_{leak} recorded corresponding to each transport curve above. **(g)-(i)** Hysteresis measurements of field effect and *IV* curves for normal MoS_2 FET, with **(j)-(l)** the gate leakage current I_{leak} recorded corresponding to each transport curve above.

Supplementary Note 1: Conventional metal contacted MoS_2 FETs

We characterized multiple samples of MoS_2 FET with conventional Au (50 nm) electrodes. As shown in Supplementary Figure 4, the typical transport behavior of *n*-type MoS_2 FET can be seen. Color map of *IV* curves as a function of gate voltages in Supplementary Figure 4c-d indicates ON states at positive and negative source-drain bias voltages (V_{ds}) at the electron side, with the hole side all turned off. Line cuts of *IV* along fixed V_g are plotted in Supplementary Figure 4e, as most of the curves slightly deviated from linear behavior, while conductance increases with increasing gate voltage. Supplementary Figure 4f plots the line cuts of transfer curves at fixed bias voltage V_{ds}. One can see typical *n*-type unipolar field effect of directly metal-contacted devices. Multi-samples confirm the same behavior, which agrees with the works previously reported [1-3].

Supplementary Note 2: Tunnel contacted MoS_2 FETs

Supplementary Figure 5 illustrates the line cuts of transfer curves at fixed bias voltage V_{ds}. Instead of the rather symmetric V_{ds} polarization with ON state only seen in the electron side for metal contacted MoS_2 FET (Supplementary Figure 4), the MoS_2 TC-FET on the same piece of MoS_2 flake, as well as in the same gate range, show strongly asymmetric V_{ds} polarization in the whole gate range. Interestingly, when the tunnel bias voltage is larger than a threshold value of about +1 V, the device start to exhibit ambipolar transfer curves, with the hole side conductivity comparable, sometimes even larger than that of the electron side.

We recorded the gate leakage current (I_{leak}) from the bottom graphite gate concomitant with transport measurement, to rule out any such influence. It is confirmed that leakage current is limited in a sub 100 pA range, compared to the measured bipolar field effect curve giving I_{ds} up to over 100 nA.

When at negative V_{ds}, the transfer curves of TC-FETs become unipolar up to the highest tested gate range (Supplementary Figure 5), but still differs from conventional FET. After the turn-on point, I_{ds} initially increases exponentially, and then enters a broad saturation plateau at higher electron doping. It is noteworthy that a shoulder toward saturation is often seen also on the electron side of the transfer curve at positive V_{ds}, such as indicated in Fig. 2b in the main text.

We find that the amplitude of I_{ds}-V_g characteristics is dependent on the area of tunnel contacts, as well as the dimensions ($L \times W$) of the channel of the TC-FET. As shown in Supplementary Figure 6, the aspect ratio of left and right TC-FETs are 4~5 times difference, and the areas of the TC contacts of them are also 4~5 times difference, which explains the resulted magnitude of ~25 times change in the I_{ds}-V_g characteristics.

To verify the reproducibility of the observed ambipolar FET behavior at finite positive V_{ds}, we fabricated multiple samples with similar geometry. All of them show in general similar curves at V_{ds} = 2 V, as illustrated in Supplementary Figure 7. We also inverted the source drain electrodes and obtained similar behavior within the gate voltage limit which keeps gate leakage negligible.

Supplementary Note 3: First principles simulations

Structural setups

In our first-principles simulations (LDA with HGH functional), in order to achieve the smallest lattice mismatch, we used 2×3 unit cells of Au atoms in (100) orientation, 2×3 unit cells of h-BN and 1×4 unit cells of MoS_2 to set up the device. The relaxed lattice structures of Au, h-BN, MoS_2 and the corresponding lattice constants in *x, z* directions are shown in Supplementary Figure 8a-c. Due to the fact that the band structure of MoS_2 is sensitive to its lattice structure, we parameterized the lattice constants of Au and h-BN solely in the *x* direction to match that of MoS_2. Because the lattice constant in *x* direction of h-BN is very similar to that of MoS_2, we

applied only a 0.9% tensile strain of h-BN in the *x* direction to make it matching with MoS_2. For the Au electrode, we need to apply a 3.4% compression strain in *x* direction to make it matching with MoS_2, which is acceptable with only slight changes in the band structures of the metal.

The leftmost and rightmost four layers of the Au electrodes in the device are determined via a separate calculation and shifts rigidly relative to each other by the external bias voltage. The rest part is the central scattering region that contains a portion of the Au electrodes, thereby establishing the bonding between the channel and the electrode, the common Fermi level, and the charge neutrality at equilibrium. The infinite open boundary problem is thereby reduced to a proper, self-consistent calculation of the charge density for the finite-sized scattering region.

The channel length of monolayer MoS_2 is set to be around 6 nm. The central scattering region including MoS_2 channel, BN tunnel contact, and Au electrode extension, is of 9.8 nm in z direction. And the system size is 0.543 nm in the periodic *x*-direction (Supplementary Figure 8b). Back gate is replaced by a uniform dielectric with dielectric constant of 7.1, and thickness of 5 nm (Supplementary Figure 8d-e).

Relaxation limits

The geometries are optimized until all residual force tolerance is smaller than 0.05 eV $Å^{-1}$. This relaxation limit follows the previous studies on the metal-MoS_2 contacts [4-7]. As mentioned in the previous section, we change the lattice constants in *x* direction of Au and h-BN to match the MoS_2 due to the sensitive influence of lattice structure on the band structure of MoS_2.

Because the lattice constant in the *x* direction of h-BN is very similar to that of MoS_2, we applied a 0.9% tensile strain of h-BN in *x* direction to make it match up with MoS_2, while a 3.4% compression strain in the *x* direction is needed for the Au electrode to match with MoS_2. Therefore, the distribution of strains in the system is mainly localized to the Au electrodes after relaxation. Although the compression strain will affect the electronic structure of Au electrode, the general metallicity cannot be changed. Therefore, the strains of Au electrodes have little effect on our

calculation about the device's electronic transport properties.

We adopted an energy cutoff of 150 Ry as in previous study of the Metal-$MoSe_2$-Metal device [8]. Calculations with larger energy cutoff (180 Ry and 200 Ry) were also tested. The calculated results such as band gap of MoS_2, transmission spectrum of normal- and tunnel-contacted device are similar to that of 150 Ry. Therefore, an energy cutoff of 150 Ry is eventually used in this work to achieve a balance between calculation efficiency and accuracy.

Tunneling barriers

To explore the effective barrier height of 2-layered h-BN, we calculated the average electrostatic potentials along y directions for each model in Supplementary Figure 9. The effective tunneling barrier height ΔV is defined as the potential barrier height above the Fermi level of the metal contact [9], indicated by the blue rectangle in Supplementary Figure 9a-b. Two tunnel barriers in series TB1 and TB2 (Supplementary Figure 9b) can be seen in the 2-layered h-BN TC-FET model in comparison with the conventional Au-MoS_2 contacted model. As can be seen from Supplementary Figure 9b, The maximum tunneling barrier height ΔV_{max} is about 4.05 eV.

Transmission and device behavior

As shown in Supplementary Figure 10a, for normal contact device, one can see there is a transmission forbidden region (1.8 eV) from -1.56 eV to 0.24 eV which is attributed to the band gap of monolayer MoS_2. Because the conduction band minimum (CBM) of MoS_2 is closer to Fermi level than that of valence band maximum (VBM), typical transport behavior of n-type FET can be seen in this normal device (shown in Supplementary Figure 4). Supplementary Figure 10b shows that when the monolayer MoS_2 is covered by 2-layered h-BN, the transmission forbidden region extends to 2.5 eV due to the associated effect of monolayer MoS_2 and 2-layered h-BN.

It is known that the calculated gap of 1.8 eV is close to the value of optical gap of

monolayer MoS_2. If one takes into account the quasi-particle excitation in the simulations, the electronic band gap will be around 2.3-2.6 eV. Limited by the huge computational expense added by quasi-particle calculations, in this work we used conventional LDA functions without quasi-particle corrections. However, this should not affect the general transport behaviors simulated, as have been widely adopted and shown in previous studies [5, 10-11], where the LDA has been proven to yield rather accurate band structures and transport properties of monolayer MoS_2 or other TMDCs.

The projected local density states (PLDOS) at V_{ds}=+1 V and at neutral doping in normal-contacted device is shown in Supplementary Figure 10d. It is clearly seen that, different from those shown in the main text, the normal-contacted devices are subjected to the conventional picture of Fermi level pinning, and the DOS bending, where a strong Schottky barrier plays a significant role in the electron transport. Even though the model of simulation is simplified compared to real experimental conditions, the results listed here qualitatively support the observed phenomena of rectifying behavior in tunnel-contacted MoS_2 FETs.

Further discussions on the IV curves in MoS_2 TC-FETs

We calculated the *I-V* characteristics of 1-layered h-BN TC-FET model, in which the calculated current density is 2-3 orders of magnitude higher than the 2-layered model as shown in Supplementary Figure 11a. However, the rectifying behavior was not found under gate voltages of, for example, V_g=0 V. We notice that, experimentally, the chance to have exfoliated monolayer h-BN is extremely low, and the thinnest limit used in this work is 2-layered h-BN. Nevertheless, chemical vapor deposition (CVD) grown mono layer h-BN has been reported previously [12]. Their experimental observation showed no rectifying behavior when CVD grown monolayer h-BN is used as a spacing layer, probably due to a large amount of defects existing in the CVD h-BN.

On the other hand, Fig. 3i in the main text of our manuscript shows the *I-V* characteristics of 2-layered h-BN model at V_g=3 V. One can see the current densities

at bias voltage higher than 0.8 V show a saturation tendency, which is in good agreement with the experimental result. We further plot the intensity at higher voltage and the saturation of the current can be still observed up to 1.2 V, which can be found in Supplementary Figure 11b. We expect current saturation in even higher bias voltages, although calculation difficulty (poor convergence) at far from equilibrium condition prohibits us from further simulation.

Supplementary Note 4: Cut-off frequency of the MoS_2 TC-FETs

It is of importance to have an idea on the upper limit of frequency when the TC-FETs are still operating. During the measurement, output waveform was monitored in real time with an oscilloscope, while sweeping the frequency of the input sinusoidal waves. One can see in Supplementary Figure 12 that when the input wave frequency is reaching the order of 20 kHz, output curve starts to be distorted. In the mean time, de-phasing between input and output waves also starts to show above 20 kHz. We therefore define 20 kHz as a cut-off frequency of our MoS_2 TC-FET devices.

It is noteworthy that in a device directly fabricated on SiO_2 with the Si gate heavily doped, the parasitic capacitance between electrodes (typically over 100×100 μm^2) and the back-gate could be the limitation of the bandwidth of working frequency. To reveal the physical cut-off frequency, one has to fabricate the devices on insulating substrates that are specially designed for high frequency tests.

Supplementary Note 5: Hysteresis measurements in the MoS_2 normal and TC-FETs

The use of tunnel-thin h-BN layer intercalated between the S/D contacts and MoS_2 channel induces an additional interface in the device. It is therefore important to

clarify if extra hysteresis is caused either in the field effect or *IV* curves. Here we show side-by-side comparison between TC-FET and normal MoS_2 FET their hysteresis measurements in Supplementary Figure 13, with a sweeping rate of 20 mV s^{-1} for each curve.

It can be seen that the TC-FET shows as small hysteresis as that of normal MoS_2 FET, in both field effect curves and *IV* curves. This can rule out, in future applications, the reliability issues which could be caused by charge trapping between the h-BN and MoS_2 interfaces.

Supplementary Methods

It is known that in the fabrication process toward resist-free pristine van der Waals heterostructures [13-15], one of the limitations is its stacking sequence: a thick enough h-BN has to be picked up first by polymer (Propylene-Carbonate, PPC, for example) to serve as a top layer. Further layers, including various layered 2D materials, will be dry-picked one after another by van der Waals force with usually all smaller in size compared to the top one [16]. Modified recipe (with Polycarbonate, PC) is then reported to be more versatile in terms of arranging the stacking sequence, disregarding the size of the to-be-stacked flakes [17]. However, when the top layer is too thin (less than 5 layers), ruptures and wrinkles increase significantly, thus reduce the quality of the final device. In particular, PC leaves more contaminations than PPC, when it is in need of flat, ultra-thin, and pristine stack, the conventional stacking technique fails, as indicated in Supplementary Figure 1.

In this work, we developed a high sample yield reverted van der Waals (vdW) deposition technique, thorough which vdW stacking with ultra-thin pristine top layer can be achieved readily. We first pick up raw layered materials in a reverted order as to the aimed final stack. All layers are collected by the first relatively thick (15-20 nm) h-BN (Supplementary Figure 2a) to avoid direct contact with PPC.

Supplementary Figure 2 illustrates a typical workflow of our process. Opposed

to the often-applied stacking method in a top-down sequence [16], we first pick up raw layered materials in a reverted order (bottom-up sequence) as the aimed final stack (Supplementary Figure 2c). A graphite gate with thickness of about 4~6 nm is used, to improve the gate efficiency and uniformity (Supplementary Figure 2d). Few-layered MoS_2 is sandwiched by a thick (~10 nm) BN and thin (2-4 layer) BN, respectively, with the resulted top later picked up lastly (Supplementary Figure 2e). When the whole stack is collected, the PPC stamp will be flipped upside down, peeled off with care from the PDMS substrate, and slowly landed onto a hot plate of about 100 ^{o}C (Supplementary Figure 2f-h). At this stage, the stack will be floating on the PPC film, which can be completely evaporated in a vacuum annealer at 350 ^{o}C for around 20 min. A typical optical image of such final stack with ultra-thin top layer is shown in Supplementary Figure 2i.

Followed by standard lithography and metallization, the final device is indicated in the schematic in Fig. 1e-f in the main text. MoS_2 flake is half covered by 2-4 layer h-BN, and 20 nm thick Au electrodes are deposited onto the stack, forming conventional direct contacts and tunnel contacts, respectively. Atomic force micrograph scan confirms that devices made in this method are of high quality top tunnel layer with roughness smaller than 0.2 nm, and without obvious wrinkles nor ruptures over 10×10 μm^2 area (Supplementary Figure 3).

Supplementary References

1 Radisavljievic, B., Radenovic, A., Brivio, J., Giacometti, V. & Kis A. Single-layer MoS_2 transistors. *Nat. Nanotechnol.* **6**, 147 -150(2011).

2 Cui, X. *et al*. Multi-terminal transport measurements of MoS_2 using a van der Waals heterostructure device platform. *Nat. Nanotechnol.* **10**, 534-540 (2015).

3 Kim, S. *et al*. High-mobility and low-power thin-film transistors based on multilayer MoS_2 crystals. *Nat. Commun.* **3**, 1011 (2012).

4 Kang, J. H., Sarkar, D., Liu, W., Jena, D. & Banerjee, K. A computational study of metal-contacts to beyond-graphene 2D semiconductor materials. *IEEE Int. Electron Dev. Meet.* 407-410 (2012).

5 Kang, J. H., Liu, W., Sarkar, D., Jena, D. & Banerjee, K. Computational study of metal contacts to monolayer transition-metal dichalcogenide semiconductors. *Phys. Rev. X* **4**, 031005 (2014).

6 Leong, W. S. *et al*. Low resistance metal contacts to MoS_2 devices with nickel-etched-graphene electrodes. *ACS Nano*. **9**, 869 -877(2015).

7 Pandey, M. *et al*. Defect-tolerant monolayer transition metal dichalcogenides. *Nano Lett.* **16**, 2234-2239 (2016).

8 Çakır, D. & Peeters F. M. Dependence of the electronic and transport properties of metal-$MoSe_2$ interfaces on contact structures. *Phys. Rev. B* **89**, 245403(2015).

9 Lee, G. H., Kim, S., Jhi, S. H. & Lee, H. J. Ultimately short ballistic vertical graphene Josephson junctions. *Nat. Commun.*, **6**, 6181 (2015).

10 Chang, C. H., Fan, X. F., Lin, S. H. & Kuo, J. L. Orbital analysis of electronic structure and phonon dispersion in MoS_2, $MoSe_2$, WS_2, and WSe_2 monolayers under strain. *Phys. Rev. B* **88**, 195420 (2013).

11 Gong, C., Colombo, L., Wallace, R. M. & Cho K. The unusual mechanism of partial Fermi level pinning at metal-MoS_2 interfaces. *Nano Lett.* **14**, 1714-1720 (2014).

12 Wang, J. *et al*. High mobility MoS_2 transistor with low Schottky barrier contact by using atomic thick h-BN as a tunneling layer. *Adv. Mater.* **28**, 8302–8308 (2016).

13 Geim, A.K. & Grigorieva, I. V. Van der Waals heterostructures. *Nature* **499**, 419 -425 (2013).

14 Novoselov, K. S., Mishchenko, A., Carvalho, A. & Castro Neto A. H. 2D materials and van der Waals heterostructures. *Science* **353**, aac9439 (2016).

15 Dean, C. R. *et al*. Boron nitride substrates for high-quality graphene electronics. *Nat. Nanotechnol.* **5**, 722 -726 (2010).

16 Wang, L. *et al*. One-Dimensional Electrical Contact to a Two-Dimensional Material. *Science* **342**, 614-617 (2013).

17 Zomer, P. J., Guimaraes, M. H. D., Brant, J. C., Tombros, N. & van Wees, B. J. Fast pick up technique for high quality heterostructures of bilayer graphene and hexagonal boron nitride. *Appl. Phys. Lett.* **105**, 013101(2014).
